# Evaluation of image quality in pediatric portable chest radiographs using AI-based noise reduction and edge enhancement

**DOI:** 10.1007/s11604-025-01887-2

**Published:** 2025-10-10

**Authors:** Atsuko Fujikawa, Shin Matsuoka, Yuki Saito, Shoko Arizono, Kosei Nakamura, Aya Kato, Takao Tanuma, Hidefumi Mimura

**Affiliations:** 1 Department of Diagnostic and Interventional Radiology, St. Marianna University School of Medicine, 2-16-1 Sugao, Miyamae-ku, Kawasaki City, Kanagawa ZIP 216-8511 Japan; 2 Graduate School of Medical Science, Kanazawa University, 5-11-80 Kodatsuno, Kanazawa, Ishikawa 920-0942 Japan

**Keywords:** Neonatal chest radiography, Artificial intelligence, Noise reduction, Edge enhancement, Image quality assessment, Low-dose imaging

## Abstract

**Purpose:**

To evaluate the image quality of pediatric portable chest radiographs processed using a deep learning–based noise reduction (NR) algorithm implemented in clinical radiography systems, which is designed to reduce image noise without altering radiation dose, both alone and with edge enhancement.

**Materials and methods:**

This retrospective visual grading analysis included 101 pediatric patients (median age: 33 days; median weight: 2844 g) who underwent portable chest radiography. Each image was processed using four techniques: (1) standard (no processing), (2) edge enhancement only, (3) NR only, and (4) NR with edge enhancement. Image quality was assessed using five criteria: visualization of proximal bronchi, small peripheral airways, vertebrae, image noise, and overall image quality. In an anonymous, randomized review, two pediatric radiologists rated each criterion using a 5-point Likert scale. Statistical comparisons were conducted between processing methods.

**Results:**

Images processed with NR and edge enhancement (NR + /Filter +) achieved the highest mean scores across all criteria. Structural visibility—particularly of small peripheral airways, proximal bronchi, and vertebrae—showed significant improvement with edge enhancement (*p* < 0.0001). No significant difference in image noise was observed between NR-only and NR + /Filter + groups (*p* = 0.482).

**Conclusion:**

AI-based noise reduction significantly improves image quality by reducing noise. Although edge enhancement does not further suppress noise, it improves the visibility of delicate anatomical structures. This combined approach may enhance diagnostic confidence in neonatal chest radiography, particularly under low-dose conditions.

## Introduction

Minimizing radiation exposure is critical in pediatric imaging, particularly in portable radiography. Consequently, low-dose imaging protocols are widely adopted in clinical practice. However, reduced radiation doses inherently increase image noise, which can obscure anatomical structures and compromise diagnostic accuracy.

Recent advances have seen the integration of artificial intelligence (AI)-based noise reduction techniques across various imaging modalities, aiming to enhance image quality while maintaining or even reducing radiation dose levels [1, 2]. One commercially available technique involves AI-based noise reduction algorithms integrated into radiographic systems, enabling effective noise suppression while retaining anatomical detail and image texture [3].

Traditionally, edge enhancement filters have also been used in radiography to improve the visibility of anatomical structures and pathological findings [4]. Given these parallel developments, this study aimed to evaluate the visual image quality of pediatric portable chest X-rays processed with NR alone and with edge enhancement. The goal was to determine the relative diagnostic utility of each processing approach.

## Objective

The objective of this study was to evaluate the visual image quality of pediatric portable chest radiographs processed with a commercially implemented AI-based noise reduction technology and to assess the additional diagnostic value of edge enhancement applied after noise reduction. Specifically, we aimed to determine how each image processing method influenced the visibility of anatomical structures and the overall clarity of images obtained under dose settings commonly used in routine clinical pediatric imaging.

## Materials and methods

### Study design

This study is a visual image quality assessment of pediatric portable X-ray images processed with an AI-based noise reduction technique commercially implemented in radiographic systems. To evaluate the impact of this noise reduction method and the additional effect of edge enhancement on image quality, we compared four types of image processing approaches on portable X-ray images.

### Digital X-ray equipment

1. Mobile X-ray system: Sirius Star mobile tiara (FUJIFILM Corporation)
2. Wireless flat panel detector: Digital Radiography CXDI- Series (Canon Inc.)
3. Imaging control software: CXDI Control Software NE ver. 3 (Canon Inc.) Noise Reduction: Intelligent NR (INR)
4. Edge enhancement: Edge enhancement was performed using the built-in filter function of the PACS system (PSP Corporation), which is routinely used in our clinical practice.
5. Diagnostic monitor: RadiForce RX660(EIZO Corporation)

All images underwent AI-based noise reduction during acquisition before being uploaded to PACS. The edge enhancement filter was implemented at the PACS level and was available on all PACS terminals throughout the hospital, including NICU workstations, allowing neonatologists to access processed images for clinical interpretation.

### Image processing techniques

**Fig. 1:**
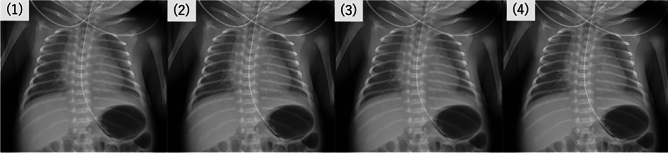
Portable chest radiographs of a 2-day-old preterm infant (34 + 3 weeks, 1,935 g). Four processing conditions are shown: (1) standard (no processing), (2) edge enhancement only, (3) noise reduction only, and (4) combined noise reduction and edge enhancement

**Fig. 2:**
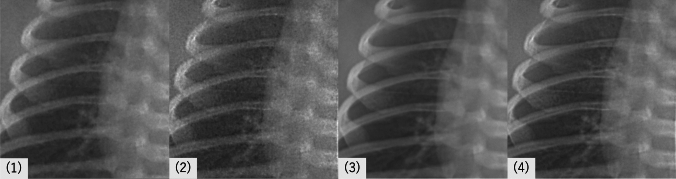
Magnified views of the lung fields from the same case shown in Fig. 1, under four image processing conditions: (1) standard (no processing), (2) edge enhancement only, (3) noise reduction only, and (4) combined noise reduction and edge enhancement. Compared with (1), image (3) demonstrates clearer depiction of pulmonary vessels due to noise suppression. Image (2) appears grainier when edge enhancement is applied without noise reduction, whereas image (4) highlights fine peripheral bronchial structures after noise reduction with additional edge enhancement

**Fig. 3:**
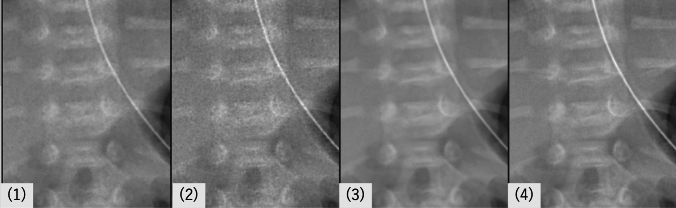
Magnified views of the vertebral region from the same case shown in Fig. 1, under four image processing conditions: (1) standard (no processing), (2) edge enhancement only, (3) noise reduction only, and (4) combined noise reduction and edge enhancement. Noise reduction improves the clarity of vertebral contours, as seen in (3), while additional edge enhancement after noise reduction in (4) further accentuates the cortical margins

Four different image processing techniques were evaluated for comparison (Fig. 1,2,3):

- Standard portable X-ray: Baseline images are obtained with the Canon DR system without additional processing.
- Portable X-ray with edge enhancement: Baseline images with an edge enhancement filter applied to improve sharpness and lesion visibility.
- Portable X-ray with noise reduction processing: Images processed using Canon’s INR software to reduce image noise while maintaining structural detail.
- Portable X-ray with noise reduction processing and edge enhancement: Images processed with both INR and an additional edge enhancement filter to evaluate the combined effect on image quality.

### Participants

The study included pediatric patients, not limited to neonates, for whom an AI-based noise reduction technique had been applied. The study population consisted of 101 patients, with 59 males and 42 females. Ages ranged from 0 to 2857 days (7.9-year-old), with a median age of 33 days (mean 223.9 days, SD 553). Body weights ranged from 457 g to 14,000 g, with a median weight of 2844 g (mean 3714.9 g, SD 3515.6 g). This broader age range resulted from the continuous collection of pediatric cases processed with the AI-based technique over a specific period.

### Image acquisition

Portable X-ray images of pediatric patients were obtained at a low radiation dose to minimize exposure, reflecting routine clinical practice in pediatric imaging. These images were processed through the four techniques outlined above, creating images for each patient under each processing condition.

The imaging parameters varied across cases: the tube voltage ranged from 50 to 70 kV (mean, 55.9 ± 9.2 kV), and the tube current–time product ranged from 0.8 to 2.0 mAs (mean, 1.26 ± 0.32 mAs). The source-to-image distance (SID) ranged from 100 to 130 cm (mean, 108.6 ± 13.6 cm). The entrance skin dose (ESD) had a mean of 29.8 μGy (SD, 7.9), with values ranging from 7.8 to 62.6 μGy.

Variation in acquisition parameters and ESD was observed due to differences in patient size and imaging conditions.

### Visual evaluation criteria—visual grading analysis (VGA)

To assess image quality, we employed a Visual Grading Analysis (VGA) approach, based on criteria adapted from the study by Smet et al. [5], which evaluated neonatal chest radiographs under various imaging conditions.

From their original set of criteria, we selected five items that were deemed most clinically relevant and feasible to evaluate in our study:

1. Visualization of the proximal bronchi—visibility and continuity of main bronchial structures.

2. Visualization of the small peripheral airways—clarity of fine peripheral airway markings.

3. Visualization of the vertebrae—delineating vertebral structures through the mediastinum.

4. Diagnostic acceptability of image noise—degree of image noise and its impact on anatomical assessment.

5. Overall image quality—comprehensive subjective assessment of diagnostic utility, including structure visibility, contrast, and artifact absence.

Each criterion was scored using a 5-point Likert scale commonly used in VGA:

1 = Criterion not fulfilled.

2 = Criterion probably not fulfilled.

3 = Indecisive whether the criterion is fulfilled or not.

4 = Criterion probably fulfilled.

5 = Criterion fulfilled.

All images were anonymized and randomly ordered to prevent observer bias. The scores from all evaluators were then averaged for statistical analysis.

### Evaluation procedure

Each radiologist independently reviewed the images in a randomized order to avoid bias. Images were rated on a structured scale (e.g., 1–5) across each criterion, and the scores were averaged to assess the relative quality of each image type. Inter-observer agreement between the two readers (A.F., 19 years of radiology experience, subspecialty in pediatric radiology; Y.S., 13 years, subspecialty in pediatric radiology) was assessed using the intraclass correlation coefficient (ICC, two-way random-effects, absolute agreement).

### Statistical analysis

Pairwise comparisons were performed using the Wilcoxon signed-rank test to determine the statistical significance of differences in image quality across the four image processing methods. This non-parametric test was selected due to the ordinal nature of the data derived from 5-point Likert scale scores. Statistical significance was defined as a *p* value < 0.05. Pairwise comparisons were performed using the Wilcoxon signed-rank test. All statistical analyses were performed using R version 4.2.2 (R Foundation for Statistical Computing, Vienna, Austria).

## Result

Inter-observer agreement between the two readers was excellent, with an ICC of 0.91 (95% CI 0.87–0.94). Overall differences among the four image processing methods were first assessed using the Friedman test, which indicated statistically significant differences for all evaluation criteria (Peripheral Airways: *χ*^2^ = 195.99, *p* < 0.0001; Proximal Bronchi: *χ*^2^ = 148.92, *p* < 0.0001; Vertebrae: *χ*^2^ = 190.12, *p* < 0.0001; Image Noise: *χ*^2^ = 216.55, *p* < 0.0001). The Visual Grading Analysis (VGA) results (Table 1) showed that image quality improved across all evaluation criteria with edge enhancement and noise reduction, particularly in the NR +/Filter + group, where visibility of small peripheral airways and vertebrae markedly increased. Image noise scores were highest in both NR + groups.

**Table 1:** Mean visual grading analysis (VGA) scores by image processing method

Region of evaluation	NR −/Filter −	NR −/Filter +	NR +/Filter −	NR +/Filter +
Small peripheral airways	3.436	3.881	3.97	4.644
Proximal bronchi	3.594	3.941	4.05	4.456
Vertebrae	3.74	3.95	4.3	4.73
Image noise	4.09	4.9	4.93	4.95

*Abbreviations used for the four image processing types are as follows: *NR* + */Filter* + Images processed with both noise reduction and edge enhancement; *NR* + */Filter − *Images processed with noise reduction only; *NR −/Filter* + Images processed with edge enhancement only; *NR −/Filter − *Standard images without additional processing

Pairwise statistical comparisons between processing groups (Table 2) demonstrated that NR +/Filter + showed statistically significant improvements (*p* < 0.0001) over all other groups in terms of anatomical structure visibility, including small peripheral airways, proximal bronchi, and vertebrae. These results indicate that edge enhancement provides additional structural clarity benefits even after an AI-based noise reduction technique has effectively reduced noise.

**Table 2:** Statistical comparison of image quality scores: pairwise *p* values between processing groups. Each *p* value represents a pairwise comparison between image processing groups using the Wilcoxon signed-rank test. *p* Value < 0.05 was considered statistically significant

Region of evaluation	Comparison	NR −/Filter −	NR −/Filter +	NR +/Filter −	NR +/Filter +
Small peripheral airways	NR −/Filter −	–	0.025	< 0.0001	< 0.0001
	NR −/Filter +	0.025	–	< 0.0001	< 0.0001
	NR +/Filter −	< 0.0001	< 0.0001	–	< 0.0001
	NR +/Filter +	< 0.0001	< 0.0001	< 0.0001	–
Proximal bronchi	NR −/Filter −	–	0.030	< 0.0001	< 0.0001
	NR −/Filter +	0.030	–	< 0.0001	< 0.0001
	NR +/Filter −	< 0.0001	< 0.0001	–	< 0.0001
	NR +/Filter +	< 0.0001	< 0.0001	< 0.0001	–
Vertebrae	NR −/Filter −	–	0.028	< 0.0001	< 0.0001
	NR −/Filter +	0.028	–	< 0.0001	< 0.0001
	NR +/Filter −	< 0.0001	< 0.0001	–	< 0.0001
	NR +/Filter +	< 0.0001	< 0.0001	< 0.0001	–
Image noise	NR −/Filter −	–	< 0.0001	< 0.0001	< 0.0001
	NR −/Filter +	< 0.0001	–	0.024	< 0.0001
	NR +/Filter −	< 0.0001	0.024	–	0.482
	NR +/Filter +	< 0.0001	< 0.0001	0.482	–

*NR* Intelligent noise reduction; *Filter* Edge enhancement

In contrast, when comparing image noise scores, significant differences were found between groups with and without noise reduction applied (all *p* < 0.0001), confirming the effectiveness of the noise reduction technique in suppressing image noise. However, there was no significant difference between NR +/Filter − and NR +/Filter + (*p* = 0.482), suggesting that edge enhancement does not further reduce noise once noise reduction has been applied.

These findings demonstrate that while the AI-based noise reduction technique alone achieves excellent noise suppression, the addition of edge enhancement improves the diagnostic visibility of fine anatomical details without compromising noise characteristics.

In additional exploratory analyses, the potential influence of patient age and body weight on image quality was evaluated. Spearman’s rank correlation showed no significant association between age and image quality (*ρ* = 0.08, *p* > 0.05). Kruskal–Wallis tests confirmed no significant differences among age groups, except for NR −/Filter + (*p* = 0.017). For body weight, a moderate positive correlation was observed with image quality scores (*ρ* = 0.37, *p* < 0.001).

## Discussion

This study confirms that combining advanced noise reduction techniques with traditional edge enhancement can significantly improve image quality in low-dose X-ray imaging for neonates. Such improvements may facilitate better diagnostic accuracy in a vulnerable population.

Recent advances in image processing have focused on denoising methods that enhance visual quality while preserving image quality. Deep learning-based approaches, such as those combining deep image prior with image fusion techniques, have effectively suppressed noise and improved perceptual image quality [1, 2]. Our study also improved image quality, likely due to efficient noise reduction.

The AI-based noise reduction technique substantially suppressed image noise across all evaluations. The lack of a statistically significant difference in noise scores between NR +/Filter − and NR +/Filter + groups (*p* = 0.482) suggests that the noise reduction alone may achieve optimal noise suppression. Further post-processing with edge enhancement does not contribute additional benefits from a noise perspective. This finding underscores the effectiveness of the AI-based noise reduction technique as a standalone denoising method [3].

However, our results also demonstrate that adding edge enhancement after noise reduction processing significantly improves the visualization of critical anatomical structures, including small peripheral airways, proximal bronchi, and vertebrae (all *p* < 0.0001). These improvements likely result from enhanced edge definition and local contrast, particularly valuable in neonatal imaging where subtle anatomical details are crucial [4]. Therefore, while edge enhancement does not further reduce noise, it provides diagnostic value by improving structural clarity. The significant overall differences among the four image processing methods, confirmed by the Friedman test, further underscore the substantial impact of processing choices on perceived image quality. Both AI-based noise reduction and edge enhancement contributed meaningfully to these improvements, with their combined application achieving the best results.

Once such effective denoising is achieved, further dose reduction may be considered. However, excessive reduction in radiation dose may compromise the diagnostic image quality required for accurate interpretation. As Kalra et al. emphasized, dose optimization should always follow the ALARA (As Low As Reasonably Achievable) principle, which requires minimizing exposure while ensuring that diagnostic quality is not lost [6]. The ICRP further emphasizes the importance of Diagnostic Reference Levels (DRLs) in medical imaging as practical tools to guide such optimization efforts [7]. Nevertheless, as Gislason-Lee noted, NICU-specific DRLs are still lacking, and variability in ALARA implementation across institutions can result in underexposure and inconsistent image quality [8]. Therefore, careful and evidence-based adjustment of imaging protocols is essential to maintain a proper balance between radiation safety and diagnostic efficacy.

These findings suggest that edge enhancement may enhance structural clarity without adversely affecting noise levels after effective denoising.

Exploratory analyses further indicated that patient age had minimal influence on image quality, with only a weak trend toward higher scores in older children for NR −/Filter +. By contrast, body weight showed a moderate positive correlation with image quality scores (*ρ* = 0.37, *p* < 0.001). This relationship likely reflects the greater image noise observed in smaller patients, particularly neonates undergoing low-dose imaging. These smaller patients appeared to benefit the most from AI-based noise reduction, which effectively compensated for their higher baseline noise levels. In larger patients, image quality tended to be more stable due to improved signal-to-noise ratio and exposure stability, which may explain the overall positive correlation between body weight and image quality. Given the skewed distribution toward NICU cases, however, the clinical relevance of the observed age-related trend is likely limited.

These results highlight the importance of tailoring image processing strategies to clinical needs: INR is highly effective in reducing noise, and edge enhancement adds complementary value by improving visualization of fine anatomical details. Together, these techniques contribute to better diagnostic confidence in neonatal chest radiography performed under low-dose conditions.

These processing options are readily available in routine practice, as noise reduction is applied before PACS upload, and edge enhancement can be accessed on all PACS terminals, including NICU workstations.

## Limitations

This study has several limitations. First, the sample size was relatively small, which may have limited the statistical power of comparing processing methods. Second, the evaluation focused on visual image quality rather than diagnostic performance. Specific pathological conditions were not assessed, and the effect of image processing on diagnostic accuracy in clinical settings remains uncertain. Future studies should investigate the diagnostic utility of these processing techniques in specific disease contexts to establish their clinical relevance.

## Conclusion

Integrating AI-based noise reduction with edge enhancement improves pediatric portable X-ray image quality by effectively reducing noise and enhancing structural visibility. While noise reduction alone suppresses image noise, edge enhancement contributes additional diagnostic value, particularly in visualizing fine anatomical details. This combined approach is therefore especially valuable in clinical pediatric settings where diagnostic precision is essential.
